# Supplementary material for: Indoleamine-2,3-Dioxygenase in Thyroid Cancer Cells Suppresses Natural Killer Cell Function by Inhibiting NKG2D and NKp46 Expression via STAT Signaling Pathways
Source: J Clin Med. 2019 Jun 12;8(6):842. doi: 10.3390/jcm8060842 (PMC6617210; doi:10.3390/jcm8060842)
Supplement: Supplementary file 1 [file jcm-08-00842-s001.pdf]

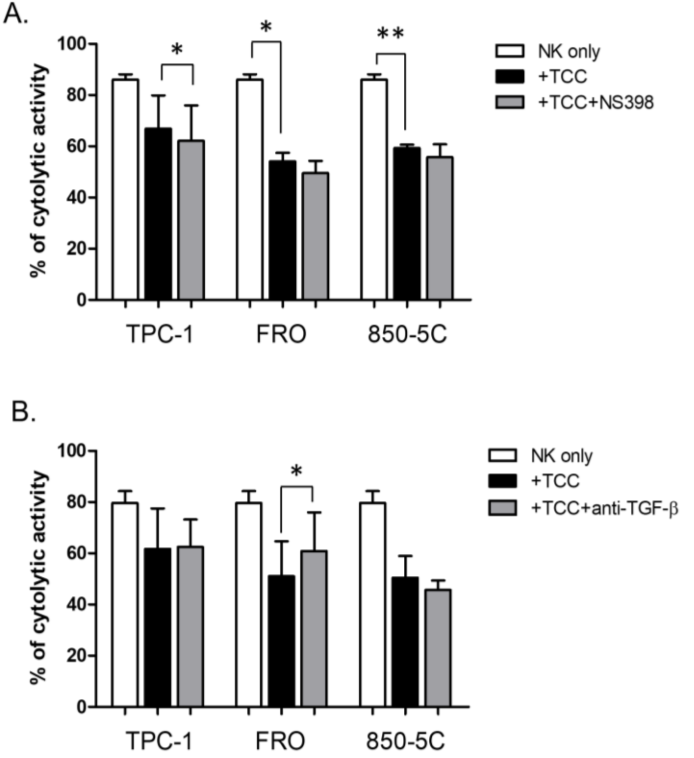


**Supplementary Figure 1.** Effect of PGE2 and TGF-β blockade on restoring NK activity in the co-culture system. (**A**) NK cells were cultured for 24 h with IL-2 either alone (NK only, white) or co-culture with indicated thyroid cancer cells (TCC). NK/thyroid cell co-cultures were set either in the absence (black) or in the presence (gray) of inhibitor of PGE2, NS398 at 2 μM. (**B**) NK/thyroid cell co-cultures were set in the presence of anti-TGF-β (gray). K562 cells were used as target cells and the effector:target (E:T) ratio was 10:1. Bars represent mean ± SD of three independent experiments.

A.

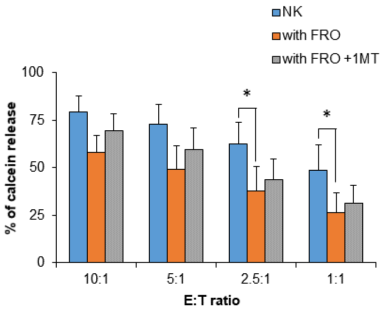


B.

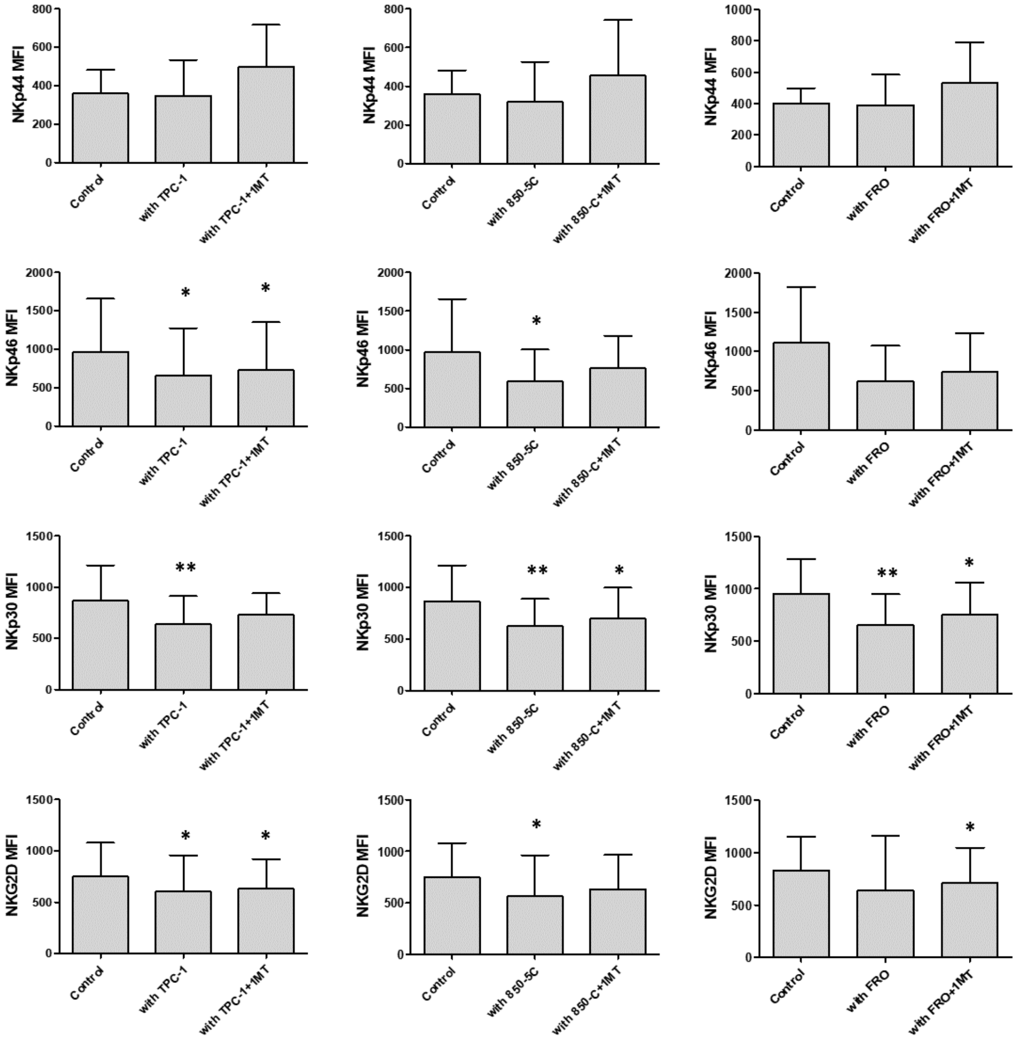


**Supplementary Figure 2.** IDO inhibitor (1MT) partially restores NK cell activity and NK receptor expressions reduced by thyroid cancer. (**A**) NK cells were cultured for 24 h with IL-2 either alone (blue) or with FRO thyroid cancer cells. NK/FRO cell co-cultures were set either in the absence (orange) or in the presence (gray) of IDO inhibitor. Bars represent mean ± SD of five independent experiments. (**B**) Expressions of NK receptors, including NKp44, NKp46, NKp30, and NKG2D were detected by flow cytometry. Graphs represent mean values of MFI for five independent experiments of receptors. Statistical analyses were performed using the paired two-tailed Student's t-test. * $P < 0.05$ and ** $P < 0.01$.

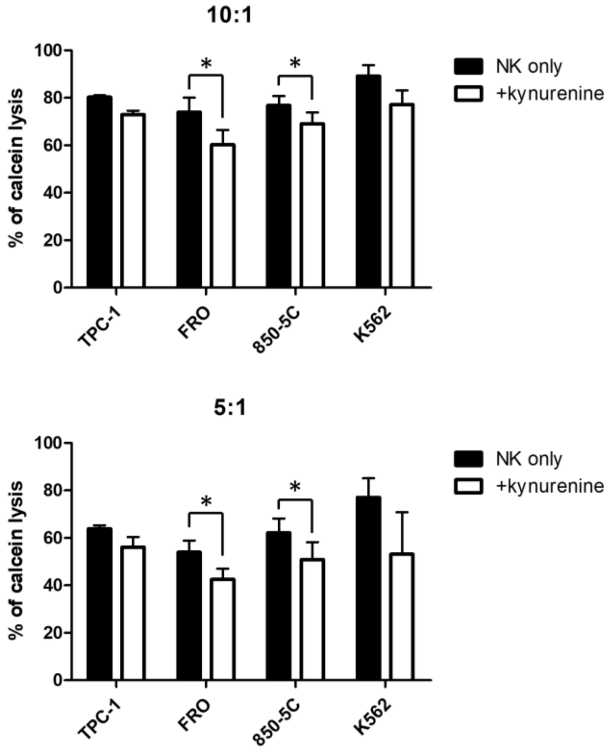


**Supplementary Figure 3.** Cytolytic activity of NK cells treated with kynurenine against thyroid cancer cells (**A**) NK cells were cultured for 24 h with IL-2 either in the absence (NK only, black) or in the presence of kynurenine 0.5 mM (white). TPC-1, FRO, 850-5C, and K562 cells were used as target cells at effector:target (E:T) ratios of 10:1 and 5:1. Bars represent mean ± SD of three independent experiments. Statistical analyses were performed using the paired two-tailed Student's t-test. * $P < 0.05$.

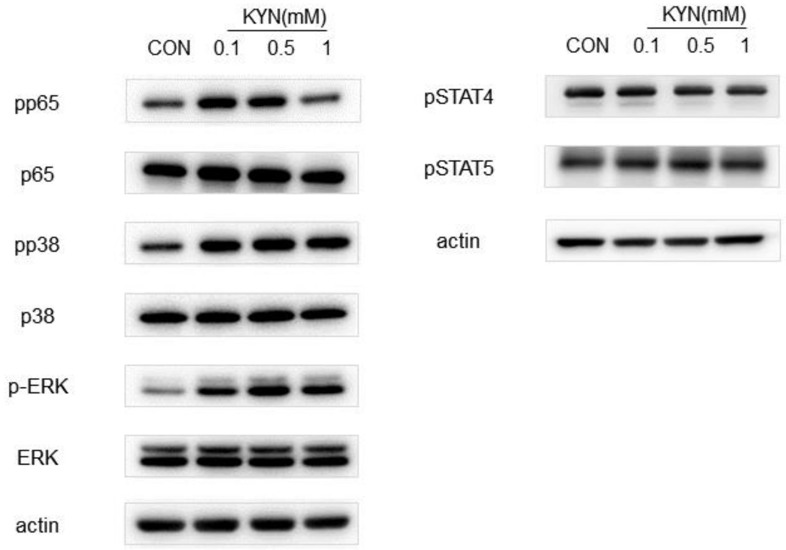


**Supplementary Figure 4.** Western blot analysis of the activation of other signals related to NK activity. Phosphorylation of pSTAT4, pSTAT5, p65, p38, and ERK signals were analyzed by Western blotting after kynurenine treatment in NK cells. β-actin served as a loading control.

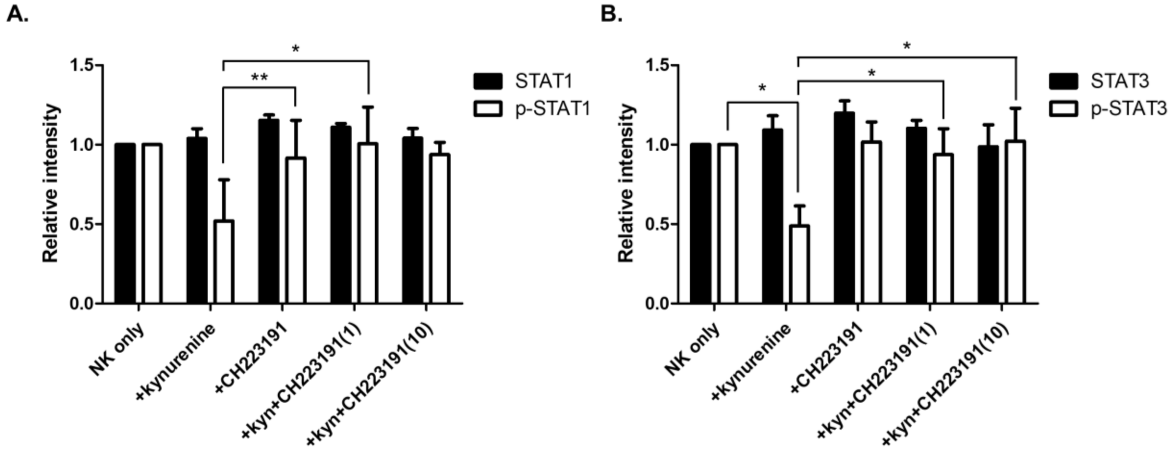


**Supplementary Figure 5.** Kynurenine inhibits STAT signals in NK cell through AhR. **(A)** Phosphorylation of STAT-3 and 1 signal were examined by Western blotting in NK cells treated with the indicated agent. Relative intensity is defined as the intensity of the target protein normalized to β-actin. Bars represent mean ± SD of three independent experiments. Statistical analyses were performed using the paired two-tailed Student's t-test. * $P < 0.05$ and ** $P < 0.01$.

NKp46 promoter

```
>hg38_knownGene_uc061ctw.1 range=chr19:54904663-54906187 5'pad=0 3'pad=0
strand=+ repeatMasking=none

gttgggactacaggcatgtgccaccacacccggctaattttgaatttttagtagagacagggtttctgcatgttg
gtcaggctggtctcgaactcccgacctaaggtgatccacctgcctcagcctcccaaagtgctgggatgacaggcc
tgagccaccgtgtctggccagtatttggtttctgtttctgtgttaactcgcttaggataatggcctctagctgc
atccatgttgctgcaaaggacataatcttgtgatttttcaaggctgtatagcgttctgtggtgtatacatatcac
attgtctttatccagtccacctctgatgggacctgggtggattccatgtcttcactattgtgaatcctgctgcaa
tgaacatacaagtgcatgtgtctttttggtagaatgatttattttcctttggctatatacccagcgatgggattg
ctgggctgaatggtaactctgtttgtagttctctgaaatatctccaaaccaaactgctttccacagtggctgaac
taatttacacccaccaacagtgtataagtgtccccttgctccacaatctcaccagcatctgttaatttctggct
tttcagtaatggccattctgactggtgtgagatggtattgttgagggataatttaggaatcagagagaccgaggg
gttgaggaggatttattattattattatttaggtgcaccggccccagtcagattaacatccaaaaagactga
ggctcgaacagagagtccggttacctttaagcatttgtggggttgggggagatctgtgcagggggaagcatat
tacagaagcaagaaacaaaggcagttattcaattgagacatgcatcacattattccttacttttcaagaaaaata
tgttttacgacttgaggttatcctgtctagtgaccttgcagccgcacggcaagagaaacagggtcttcacaatgc
ctgggaaagggagagataaggctcactagccacagacagaaaaacaggcagttcatgtttaaaggactccacctc
tttctcttcctcggggggaactgggtttcttaaatacaactgagttttgtttacacattctgtaatttctttt
aattcctgttccagtatctcactgtgaaactccctatgtttttatacgattctcaggggggtttcctctgggcatg
attgggcacaacttcccacagtcagctctgggtacgacctccacattgcagaattgagaagttgacccagaaatg
cattttgggctgagcagacaattgtcagagttgctggctagaccacagatgtgtcagagggaccacggcctttct
gtaagctcatggtcagaggcggaggggagttgtgaacgttctgatgaaagcagtcaacgtgaaagcgctctggtg
atgggcgctggtgctcacccaccacttcctgtgtatctatctccctggcccgcccggctcagtccccactgctca
GCACTAGGCCGGCAGAATCTGAGCG
```

NKG2D promoter

```
>hg38_knownGene_uc009zhj.4 range=chr12:10388811-10391518 5'pad=0 3'pad=0
strand=- repeatMasking=none

aacatattttcttcaacttgttttatattcacctatcttgtttaattttgaatttgttttatacttattcatttt
atatcacctaaactaaaaagtaatttccagctgggtgtggtggctcacgtctgtaatctcagcacattgggaggc
tgaggcaggaggatcacctgaggtcaggagttcgagaccagcctgactaacatggtgaaacctcgtctctactag
aaatacaaaaattagccaggtgtggtggcacatgcctgtaatcccagttattctggaggctgagacaggagaatt
gcttgaacccaggtggcggaggttgcagtgagccgagatcgccccgttgcactccagcctggacaacaagagtga
aactccatctcttaaaaaaaaaaaaaaaaaaagtaattaatttccatgtgggcaaaaactttcttttggattgtt
tactttgacattctcaagatctagaaaagtgcatagcaaatagcattcgctaaagaaaaaaaatgttgaataaat
ttattgtccaactttaacacatcaccacatgaaagctgtttccttccttatatttcagagttttaagattaata
gctttggtttttgttaaaatgttataatgtgttcatcttatattactttatatgatgtcttatgttagtattta
aattaataaacataaaatatatatttagcttatcttgttcattggtatcaattcatatattattcttatgtatac
tggggtatatatttaagtttctaattctcctgcatatgatgtagatatgacagagatgcaatttcatgtttttta
tattaaaatttaaaaattcccaggactaatttttgtaacttaaaggccagtgtgatagtaacccaggacaaatt
attgaataggagctaatttcccactttatctgcaatgttaccttatattttttaatctatgcacatgactttttc
ttgtctgtatttttacccttctcaatttgtctatctatgccccacatgccactttgcctttattaatacggtttt
aacataggtcttactatctagtaaaacaaggctcccacatttcttcatcttcaattgtgactttcctagtcttg
acttttgtaatttaatgtacatgtagaattctgctttgtaagttctacaacaaaacctgaggtattaactggaat
tgaattgaatctctaaaatatttaggattaaatggcatcatttaaattcatcattttcaaatcctaaaatgttat
ttaaaataatggcaaaactacaattactttgcaccaacctaacatttatccaacaatgggtggtcttaactctt
ttaccaaattaacaatatttttgtagtgataaggcggaagtactgtggtttatgtttttccacaggaagatgaga
ttataatatttgtcacatctttctcaaagatagaaattcactaagtatctccactttcaattctagatcaggaac
TGAGGACATATCTAAATTTTCTAGTTTTATAGAAGGCTTTTATCCACAAGAATCAAGATCTTCCCTCTCTGAGCA
GGAATCCTTTGTGCATTGAAGACTTTAGATTCCTCTCTGCGGTAGACGTGCACTTATAAGTATTTG
```

**Supplementary Figure 6.** Receptors of NK cells promoter sequences. Promoter sequences of NK receptors and STATs motif (underlined). Promoter sequences were obtained through UCSC page (https://genome.ucsc.edu/). The predicted binding motif site in the sequences was determined using a program FIMO.
